# Analysis of the IDDM Candidate Gene *Prss16* in NOD and NON Mice

**DOI:** 10.1080/10446670310001593497

**Published:** 2002-12

**Authors:** Saijai Cheunsuk, Tom Hsu, M. Eric Gershwin, Christopher L. Bowlus

**Affiliations:** 1 Division of Gastroenterology Department of Internal Medicine University of California Davis Davis CA 95616 USA; 2 Division of Rheumatology Allergy and Clinical Immunology Department of Internal Medicine University of California Davis Davis CA 95616 USA

## Abstract

The thymus-specific serine protease *Prss16* is highly expressed by the epithelial cells in the thymic cortex. It has been suggested to play an important role in the positive selection of T cells through the antigen presention pathway of the cortical antigen presenting cells. Recently, the gene ecoding *Prss16* has been linked to insulin dependent diabetes mellitus (IDDM) susceptibility independent of HLA-DR3 suggesting the *Prss16* may be involved in the development of autoimmune disease. Due to the similarities of the gene structure and expression pattern between the human and mouse genes, we compared *Prss16* between non-obese diabetic (NOD) and non-obese non-diabetic (NON) mice. Analysis of the *Prss16* coding region failed to identify any differences in sequence. Northern analysis and semi-quantitative reverse transcriptase polymerase chain reaction showed that the mRNA was equal in size and abundance in the two strains. *In situ* hybridization showed similar patterns of staining. Therefore, our data suggests that there is no significant different in the gene structure, transcription level, and expression pattern of *Prss16* gene between NOD and NON mice.


*Developmental Immunology*, December 2002 Vol. 9 (4), pp. 183–186

Taylor & Francis healthsciences

### Analysis of the IDDM Candidate Gene *Prss16* in NOD and NON Mice

SAIJAI CHEUNSUK[a], TOM HSU[b], M. ERIC GERSHWIN[b] and CHRISTOPHER L. BOWLUS[a,*]

[a]*Division of Gastroenterology, Department of Internal Medicine, University of California Davis, Davis, CA 95616, USA;* [b]*Division of Rheumatology, Allergy and Clinical Immunology, Department of Internal Medicine, University of California Davis, Davis, CA 95616, USA*

The thymus-specific serine protease *Prss16* is highly expressed by the epithelial cells in the thymic cortex. It has been suggested to play an important role in the positive selection of T cells through the antigen presention pathway of the cortical antigen presenting cells. Recently, the gene ecoding *Prss16* has been linked to insulin dependent diabetes mellitus (IDDM) susceptibility independent of HLA-DR3 suggesting the *Prss16* may be involved in the development of autoimmune disease. Due to the similarities of the gene structure and expression pattern between the human and mouse genes, we compared *Prss16* between non-obese diabetic (NOD) and non-obese non-diabetic (NON) mice. Analysis of the *Prss16* coding region failed to identify any differences in sequence. Northern analysis and semi-quantitative reverse transcriptase polymerase chain reaction showed that the mRNA was equal in size and abundance in the two strains. *In situ* hybridization showed similar patterns of staining. Therefore, our data suggests that there is no significant different in the gene structure, transcription level, and expression pattern of *Prss16* gene between NOD and NON mice.

*Keywords*: Thymus; *Prss16*; Serine protease; Polymorphisms; NOD; Diabetes

Type 1 or insulin-dependent diabetes mellitus (IDDM) is a polygenic autoimmune disease caused by T cell-mediated destruction of insulin-producing pancreatic β cells (Birk and Cohen, 1993; Vyse and Todd, 1996). IDDM susceptibility is most tightly associated with genes linked to the HLA region of the Major Histocompatability Complex (MHC) on human chromosome 6p21. The strongest associations reside within the *HLA- DRB1, HLA-DQA1*, and *HLA-DQB1* (class II) regions (Todd *et al.*, 1987; Nepom, 1995). However, variants in this region do not fully explain the association between the MHC and IDDM (Robinson *et al.*, 1993; Lie *et al.*, 1999a). Using transmission disequilibrium testing, Lie *et al.* have shown evidence for an IDDM susceptibility locus independent of MHC class II located telomeric of the MHC region near the *D6S2223*. The strongest candidate gene for this susceptibility locus is *Prss16*, also referred to as thymus specific serine protease (Bowlus *et al.*, 1999).

*Prss16* encodes a protein with homology to prolyl-carboxypeptidase (PCP) and is specifically expressed by cortical epithelial cells in the thymus. The pattern of expression around the thymic capsule and vessels is similar to that reported for cathepsin L, a protease involved in antigen presentation during the positive selection of T cells (Nakagawa *et al.*, 1998). Similarly, *Prss16* is believed to function in the positive selection of T cells. Defects in positive selection have been implicated in the development of autoimmunity and IDDM, in particular (Luhder *et al.*, 1998).

The purpose of this study was to evaluate *Prss16* as a diabetes susceptibility locus in the non-obese diabetic (NOD) mouse by comparison to the non-obese non diabetic (NON) mouse. Mutations that might effect the coding region, mRNA size and expression were screened for by direct sequencing, Northern hybridization, semi-quantitative real-time PCR, and *in situ* hybridization.

### MATERIALS AND METHODS

### DNA Sequencing

NON and NOD mice genomic DNA was purchased from the Jackson Laboratory (Maine, USA). Seven pairs of primers were designed using Primer Express software (Applied Biosystems, Foster City, CA) for amplification and sequencing of all 12 exons in both strains of mice (Table I). PCR reactions were performed under the following conditions: 4 ng/μl template DNA, 1 pmol/μl each primer, 1 mM dNTPs, 1.5 mM $MgCl_2$, 1X PCR Buffer, 0.02 U/μl Taq polymerase in a final volume of 50 μl. Cycling conditions were: 94°C for 3 min, 35 cycles X (94°C for 45 s, 56°C for 45 s, and 72°C for 1 min),

*Corresponding author. Division of Gastroenterology, UC Davis Medical Center, 4635 2nd Avenue, Sacramento, CA 95817, USA. Tel.: +1-916-734-0857. Fax: +1-916-456-7727. E-mail: clbowlus@ucdavis.edu

ISSN 1044-6672 print © 2002 Taylor & Francis Ltd
DOI: 10.1080/10446670310001593497

TABLE I Primers used for amplification and sequencing of *Prss16* in NOD and NON mice

| Exon(s) | Forward primer | Reverse primer | PCR product (bp) |
|---|---|---|---|
| 1,2 | EX 1/2.F (GGACAAAAGAGCAGAGTGCC) | EX1/2.R (AGAAAGCAAGGAACTGGGGT) | 446 |
| 3,4 | EX 3/4.F (GGGGGAAATCTTGGAGAAAA) | EX 3/4.R (CACAGCGGTGGATACTAACAGA) | 494 |
| 5–7 | EX5/6/7.F (GTTACCGGAAGGAACCTTGG) | EX5/6/7.R (CGCCTTCCTCCACACTCTAC) | 645 |
| 8 | EX 8.F (GAGGACAGGGAGTGAGGTTG) | EX 8.R (TTTGGCCCATTAGTCTCTGG) | 436 |
| 9 | EX 9.F (GAGAAGAGGGAACATGCAGC) | EX 9.R (TTCGGTTTGGGAGTTAGGTG) | 241 |
| 10,11 | EX 10/11.F (GAGAAGAGCAGGAGGCTGTC) | EX 10/11.R (GGAGTCAGAACTCAGCACCA) | 550 |
| 12 | EX 12.F (GGTGCTGAGTTCTGACTCCA) | EX12.R (GCCAGTCAGGATTGAGGAGA) | 440 |

72°C for 4 min. All PCR products were analyzed on a 1% agarose gel and sequenced with the same primers on an ABI 377 Sequencer using Big Dye Terminator Cycle Sequencing kit (Applied Biosystems, Foster City, CA). Sequences were aligned using Sequencher software (Genecodes, Ann Arbor, MI).

### Total RNA Isolation, Probe Labeling, and Northern Blot Analysis

Six-week-old male C57BL/6, NOD/LtJ and NON/LtJ mice were purchased from the Jackson Laboratory (Maine, USA). Total RNA was prepared from the mouse thymi using TriZol® Reagent (Life Technologies, Rockville, MD. USA) according to the manufacturer's instructions. Total RNA (10 μg) was separated on 0.8% formaldehyde agarose gel and transferred onto Hybond-N+ nylon transfer membrane (Amersham Pharmacia Biotech, Piscataway, NJ, USA). A mouse cDNA clone (Genbank Accession AI587874) was radiolabeled and hybridized as previously described. After two washes each in 2 × SSC, 0.1% SDS for 20 min at room temperature and 0.2 × SSC, 0.1% SDS for 20 min at 65°C, the membrane was exposed to the phosphor screen for 72 h at room temperature, then analyzed using the PhosphorImager (Molecular Dynamics, Sunnyvale, CA, USA).

### Thymic Stromal Cell Population Enrichment and Real Time RT-PCR

Stromal cells were isolated from the thymi of 3 weeks-old male NOD ($n = 4$) and NON ($n = 4$) mice as previously described (French *et al.*, 1997). Briefly, fresh thymus was cut into small pieces, suspended in RPMI 1640 (Life Technologies, Grand Island, NY, USA), and homogenized in a loosely fitting glass homogenizer. The cell suspension was filtered through a 100-μm pore cell strainer (FALCON) and left to sediment at 4°C for 10 min. The supernatant was separated from the sediment. The material retained by the strainer and the sediment were resuspended in cold (4°C) PBS and left to sediment at 4°C for 10 min, and the supernatant was separated from the sediment and the procedure was repeated three times. The final sediment was briefly pelleted at 800*g* and RNA was extracted using TriZol® reagent as described above.

Semi-quantitative real time PCR was performed on an ABI 7700 Sequence Detector using SYBR® Green Assays (PE Biosystems, Foster City, CA, USA.). Briefly, 500 μg of stromal RNA was reverse transcribed in a 20 μl reaction using SuperScript Reverse Transcriptase (Life Technologies) with 2.5 mM random hexamer primer (Life Technologies) according to the manufacturer's instructions. 5 μl of 1:50 dilution of cDNA reaction was used for template with primers TQ 295F (CAGCCACTGGATCC-CTTCA) and TQ 262R (TGATCATTCACCCAGTA-CCGC) in a 50 μl reaction according to the manufacturer's recommendations. Cycling consisted of: 1 × 50°C for 2 min, 1 × 95°C for 10 min, 40 × (95°C for 15 s, 60°C for 1 min). Similarly, the 18S ribosomal RNA was amplified with primers 18S.F (CTACCACATCCAAGGAAGGCA) and 18S.R (CAGACTTGCCCTCCAATGGA). All samples were performed in triplicate. PCR products were separated on a 2% agarose gel to confirm specific amplification. Quantitation was performed using Sequence Detector V1.7. A standard cDNA derived from a C57BL6 mouse thymus was used as a calibrator in each assay.

### *In Situ* Hybridization

Mouse thymi from the NOD and NON mice were sectioned at 8 μm and fixed in 4% paraformaldehyde. Sense and antisense cRNA probes were generated from the same cDNA clone used for Northern hybridization with incorporation of digoxigenin-UTP (Roche Molecular Biochemicals, Indianopolis, IN, USA). Probes were hybridized overnight at 58°C to thymic sections and washed. Following incubation with alkaline phosphatase-conjugated anti-digoxigenin antibody, slides were incubated in BCIP/NBT at room temperature for 36 h. The section were counterstained with Nuclear Fast Red (Vector Laboratories, Burlingame, CA, USA) and analyzed by light microscopy.

### RESULTS

### Genomic Structure

To determine the genomic structure of the mouse *Prss16* gene in both NOD and NON mice, genomic DNA was sequenced with seven different pairs of primers. No differences in sequence were identified between NOD and NON.

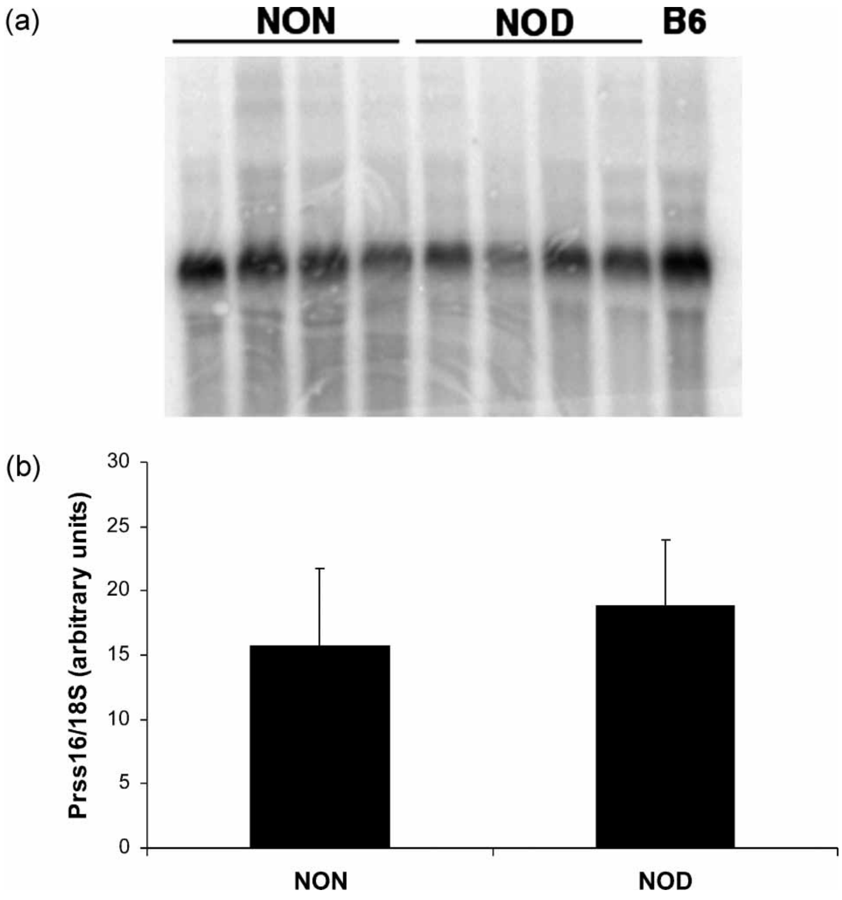

FIGURE 1 (a) Northern hybridization of *Prss16* probe to total thymus RNA from C57BL/6, NOD ($n = 4$) and NON ($n = 4$) mice. (b) *Prss16* mRNA relative to 18S ribosomal RNA in thymic stromal cells from the thymi of three weeks old male NOD ($n = 4$) and NON ($n = 4$) mice. Shown is the average and SEM for *Prss16* mRNA relative to 18S rRNA.

### *Prss16* Expression in NOD and NON Mice

Since NOD mice have abnormal thymic structure and develop the autoimmune phenotype, we investigated the thymic expression of *Prss16* in NOD and NON mice. Northern blots of NOD and NON thymus RNA were used to screen for *Prss16* mRNA level and size (Fig. 1a). No difference in size or band intensity could be detected. We also performed semi-quantitative PCR on thymic stromal cells to further investigate differences in *Prss16* mRNA (Fig. 1b). When normalized to 18S rRNA, no difference in the relative amount of *Prss16* in the thymic stromal cells from NOD and NON mice could be detected ($p > 0.70$).

We have previously shown by *in situ* hybridization that *Prss16* is much more prevalent in C57BL6 and BALB/C than NOD thymus (Cheunsuk *et al.*, 2002). However, comparison of NOD to NON thymus failed to reveal any notable difference in the pattern or density of *Prss16* staining (Fig. 2).

### DISCUSSION

Identification of genetic variants that confer susceptibility to or protection from IDDM is important to identify high-risk individuals and potential targets for preventive therapies. Several susceptibility loci have been identified including the MHC class II, the *INS* VNTR and most recently *CTLA4* (Eaves *et al.*, 1999; Ueda *et al.*, 2003). Similar influences of these loci have also been reported in NOD mice supporting its role as a model of IDDM.

In total, more than 20 non-MHC linked genes have been suggested to influence diabetes susceptibility with the MHC contributing the greatest influence (Griffiths *et al.*, 1999; Grattan *et al.*, 2002). Most of this effect has been attributed to the unusual sequence of the HLA-DQ allele in humans and H2$^{g7}$ allele in NOD mice which both encode non-aspartic acid residues at position 57 of the β chain. However, other

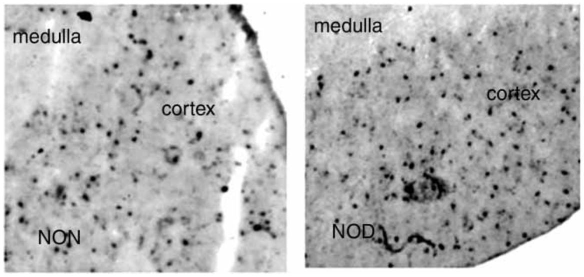

FIGURE 2 *In situ* hybridization of *Prss16* to thymi from NOD and NON mice.

genetic influences linked to the MHC have been suggested by several studies. Dissecting out the precise locus has been difficult due to the strong linkage disequilibrium in the region. However, using transmission disequilibrium tests, a clear association has been established in the region telomeric of the MHC class I region on chromosome (Lie *et al.*, 1999). Notably, the rate of transmission of the D6S2223 allele 3 was 0.2 in probands compared to the expected 0.5. In addition, this same allele is associated with susceptibility to celiac disease (Lie *et al.*, 1999b).

Several polymorphisms in *Prss16* have recently been identified including a missense mutation and a 15 base pair deletion (7548_7563del) predicted to result in a loss of 5 amino acids (Lie *et al.*, 2002). Interestingly, the 7548_7563del has an allele frequency of 0.17 and is in linkage disequilibrium with the DQA1*05–DQB1*02 haplotype. The potential association between this and other polymorphisms with IDDM is currently under investigation.

In mouse, the orthologue of *Prss16* is highly conserved with expression similarly restricted to thymic cortical epithelial cells (Cheunsuk *et al.*, 2002). In addition, *Prss16* is expressed at lower levels in autoimmune prone mice such as NOD and New Zealand Black (NZB) mice compared to C57BL6 and BALB/C mice. However, *Prss16* is not syntenic to the MHC on mouse chromosome 17. Rather, it is encoded in the comparative region centromeric of the satin (*sa*) locus on mouse chromosome 13. *Idd14* is a diabetes susceptibility locus that has been mapped to chromosome 13 linked to *D13Mit61* (McAleer *et al.*, 1995). This locus was identified by an outcross of NOD with congenic NON.H2$^{g7}$ followed by an F1 intercross. In the F2 generation, susceptibility was associated with increased NOD homozygosity and decreased NON homozygosity at *D13Mit61*.

For these reasons, we investigated *Prss16* as a possible susceptibility gene for the development of diabetes in NOD mice. Analysis of *Prss16* gene of NOD and NON mice demonstrated no mutation in the coding sequence of the NOD mice compare to that of the NON mice. Previously, we have shown by *in situ* hybridization that *Prss16* expression in NOD mice is lower compared to C57BL6 and Balb/c mice. In addition, lower expression is also noted in NZB mice which are prone to autoimmune disease as well. However, we found no difference by *in situ* hybridization between NOD and NON mice. In all strains gene expression is specific to cortical epithelial cells. Furthermore, Northern analysis and the semiquantitative PCR did not demonstrate any significant different in transcript size or abundance between the strains.

In conclusion, we have evaluated *Prss16* as a candidate gene for diabetes susceptibility in NOD mice. We were unable to detect any differences in the coding sequence, transcript size or transcript abundance. Although we cannot conclusively rule out *Prss16* as the *Idd14* diabetes susceptibility gene in the NOD mouse, our results make it highly unlikely. Interestingly, more closely linked with *D13Mit61* is cathepsin L which when deleted in mice has been shown to result in abnormal selection of CD4+ T cells and development of natural killer T cells (Honey *et al.*, 2002). Further investigation of cathepsin L as the *Idd14* susceptibility gene is warranted. It is important to note that our results do not exclude *Prss16* as a susceptibility gene in human IDDM.

### References

Birk, O.S. and Cohen, I.R. (1993) "T-cell autoimmunity in type 1 diabetes mellitus", *Curr. Opin. Immunol.* **5**, 903–909.

Bowlus, C.L., Ahn, J., Chu, T. and Gruen, J.R. (1999) "Cloning of a novel MHC-encoded serine peptidase highly expressed by cortical epithelial cells of the thymus", *Cell Immunol.* **196**, 80–86.

Cheunsuk, S., Sparks, R., Noveroske, T., Hsu, Justice, M.J., Gershwin, M.E., Gruen, J.R. and Bowlus, C.L. (2002) "Expression, genomic structure and mapping of the thymus specific protease prss16: a candidate gene for insulin dependent diabetes mellitus susceptibility", *J. Autoimmun.* **18**, 311–316.

Eaves, I.A., Bennett, S.T., Forster, P., Ferber, K.M., Ehrmann, D., Wilson, A.J., Bhattacharyya, S., *et al.* (1999) "Transmission ratio distortion at the INS-IGF2 VNTR", *Nat. Genet.* **22**, 324–325.

French, L.E., Wilson, A., Hahne, M., Viard, I., Tschopp, J. and MacDonald, H.R. (1997) "Fas ligand expression is restricted to nonlymphoid thymic components *in situ*", *J. Immunol.* **159**, 2196–2202.

Grattan, M., Mi, Q.S., Meagher, C. and Delovitch, T.L. (2002) "Congenic mapping of the diabetogenic locus Idd4 to a 5.2-cM region of chromosome 11 in NOD mice: identification of two potential candidate subloci", *Diabetes* **51**, 215–223.

Griffiths, M.M., Encinas, J.A., Remmers, E.F., Kuchroo, V.K. and Wilder, R.L. (1999) "Mapping autoimmunity genes", *Curr. Opin. Immunol.* **11**, 689–700.

Honey, K., Benlagha, K., Beers, C., Forbush, K., Teyton, L., Kleijmeer, M.J., Rudensky, A.Y. and Bendelac, A. (2002) "Thymocyte expression of cathepsin L is essential for NKT cell development", *Nat. Immunol.* **3**, 1069–1074.

Lie, B.A., Todd, J.A., Pociot, F., Nerup, J., Akselson, H.E., Ronningen, K.S., Joner, G., Dahl-Jorgensen, K., *et al.* (1999) "The predisposition to type 1 diabetes linked to the human leukocyte antigen complex includes at least one non-class II gene", *Am. J. Hum. Genet.* **64**, 793–800.

Lie, B.A., Sollid, L.M., Ascher, H., Ek, J., Akselsen, H.E., Ronningen, K.S., Thorsby, E. and Undlien, D.E. (1999) "A gene telomeric of the HLA class I region is involved in predisposition to both type 1 diabetes and coeliac disease", *Tissue Antigens* **54**, 162–168.

Lie, B.A., Akselsen, H.E., Bowlus, C.L., Gruen, J.R., Thorsby, E. and Undlien, D.E. (2002) "Polymorphisms in the gene encoding thymus-specific serine protease in the extended HLA complex: a potential candidate gene for autoimmune and HLA-associated diseases", *Genes Immun.* **3**, 306–312.

Luhder, F., Katz, J., Benoist, C. and Mathis, D. (1998) "Major histocompatibility complex class II molecules can protect from diabetes by positively selecting T cells with additional specificities", *J. Exp. Med.* **187**, 379–387.

McAleer, M.A., Reifsnyder, P., Palmer, S.M., Prochazka, M., Love, J.M., Copeman, J.B., Powell, E.E., *et al.* (1995) "Crosses of NOD mice with the related NON strain. A polygenic model for IDDM", *Diabetes* **44**, 1186–1195.

Nakagawa, T., Roth, W., Wong, P., Nelson, A., Farr, A., Deussing, J., Villadangos, J.A., *et al.* (1998) "Cathepsin L: critical role in Ii degradation and CD4 T cell selection in the thymus", *Science* **280**, 450–453.

Nepom, G.T. (1995) "Class II antigens and disease susceptibility", *Annu. Rev. Med.* **46**, 17–25.

Robinson, W.P., Barbosa, J., Rich, S.S. and Thomson, G. (1993) "Homozygous parent affected sib pair method for detecting disease predisposing variants: application to insulin dependent diabetes mellitus", *Genet. Epidemiol.* **10**, 273–288.

Todd, J.A., Bell, J.I. and McDevitt, H.O. (1987) "HLA-DQ beta gene contributes to susceptibility and resistance to insulin-dependent diabetes mellitus", *Nature* **329**, 599–604.

Ueda, H., Howson, J.M., Esposito, L., Heward, J., Snook, H., Chamberlain, G., Rainbow, D.B., *et al.* (2003) "Association of the T-cell regulatory gene CTLA4 with susceptibility to autoimmune disease", *Nature* **30**, 30.

Vyse, T.J. and Todd, J.A. (1996) "Genetic analysis of autoimmune disease", *Cell* **85**, 311–318.

